# Direct Renin Inhibition with Aliskiren Improves Ischemia-Induced Neovasculogenesis in Diabetic Animals via the SDF-1 Related Mechanism

**DOI:** 10.1371/journal.pone.0136627

**Published:** 2015-08-25

**Authors:** Ting-Ting Chang, Tao-Cheng Wu, Po-Hsun Huang, Chih-Pei Lin, Jia-Shiong Chen, Liang-Yu Lin, Shing-Jong Lin, Jaw-Wen Chen

**Affiliations:** 1 Institute of Pharmacology, National Yang-Ming University, Taipei, Taiwan, R.O.C; 2 Department of Medicine, Taipei Veterans General Hospital, Taipei, Taiwan, R.O.C; 3 Cardiovascular Research Center, National Yang-Ming University, Taipei, Taiwan, R.O.C; 4 Faculty of Medicine, National Yang-Ming University, Taipei, Taiwan, R.O.C; 5 Department of Pathology and Laboratory Medicine, Taipei Veterans General Hospital, Taipei, Taiwan, R.O.C; 6 Institute of Clinical Medicine, National Yang-Ming University, Taipei, Taiwan, R.O.C; 7 Department of Medical Research, Taipei Veterans General Hospital, Taipei, Taiwan, R.O.C; University of Edinburgh, UNITED KINGDOM

## Abstract

**Objective:**

Aliskiren is a direct renin inhibitor which is suggested to modify proangiogenic cells in addition to lower blood pressure. Given that angiogenesis is impaired in the presence of diabetes mellitus, we would like to investigate whether and how aliskiren enhances endothelial progenitor cells (EPCs) and improves ischemic-induced neovasculogenesis by an effect independent of blood pressure reduction in diabetic animals.

**Methods:**

Streptozotocin-induced diabetic mice were administered with either aliskiren (5 or 25 mg/kg/day) using an osmotic pump or hydralazine (2 or 10 mg/kg/day) given in drinking water for two weeks prior to a hind-limb ischemia surgery. Laser Doppler imaging and flow cytometry were used to evaluate the degree of neovasculogenesis and the circulating levels of EPCs, respectively.

**Results:**

In streptozotocin-induced diabetic mice, aliskiren enhanced the recovery of limb perfusion and capillary density, increased the number of circulating Sca-1^+^/Flk-1^+^ EPC-like cells, and elevated the levels of the plasma vascular endothelial growth factor (VEGF) and stromal cell-derived factor (SDF)-1α in a dose-dependent manner, whereas there were no such effects in hydralazine-treated mice. Intraperitoneal administration of anti-SDF-1 neutralizing monoclonal antibodies abolished the effects of aliskiren.

**Conclusions:**

Independent of the reduction of blood pressure, aliskiren enhanced ischemia-induced neovasculogenesis in a dose-dependent manner via VEGF/SDF-1α related mechanisms in diabetic mice.

## Introduction

Angiogenesis is impaired in the presence of peripheral arterial disease, which is frequently seen in patients with type II diabetes mellitus (DM), hypertension, or both [1]. Endothelial progenitor cells (EPCs) derived from bone marrow can be mobilized endogenously in response to vascular injury and tissue ischemia, which may contribute to vascular repair, angiogenesis, and neovascularization [2,3]. In either type 1 or type 2 DM, the number and function of EPCs can be reduced by hyperglycemia and may contribute to the development of peripheral vascular complications [4,5]. Clinically, peripheral arterial disease associated with impaired angiogenesis and neovascularization may be one of the major causes of unhealed wounds resulting in morbidity and mortality in diabetic patients.

Renin is secreted by kidney and catalyzes angiotensinogen to angiotensin I, which is the rate-limiting step in renin-angiotensin-aldosterone system (RAS). In clinical or experimental DM, RAS may be activated to induce high blood pressure and systemic vasculopathy. The RAS inhibitors such as angiotensin converting enzyme inhibitors (ACEIs) and angiotensin AT1-receptor blockers (ARBs) perform vascular protection by blocking the effects of angiotensin II [6], and/or by increasing the effects of bradykinin in the presence of DM. However, such effects may also reduce the feedback inhibition of renin synthesis by angiotensin II, resulting in a reactive rise in plasma renin [7]. Little is known about the potential effects of renin on peripheral vascular protection.

Aliskiren, a novel non-peptide and oral renin inhibitor, can directly inhibit plasma renin activity and its enzymatic effects on angiotensinogen [8]. Recently, aliskiren was shown to *in vivo* anti-inflammatory effects [9], to reduce atherogenesis [10], and to exert synergistic cardiac protective effects with ARBs on experimental myocardial infarction [11]. However, the potential mechanisms of direct vascular protection by aliskiren have not been well elucidated.

Clinically, the treatment of aliskiren is mainly for blood pressure control in hypertensive patients including those with type 2 DM. While aliskiren in combined with ACEIs or ARBs was not a useful strategy and resulted in early termination of clinical trials in patients with both diabetes and renal disease, low-dose aliskiren (150 mg/daily) in association with ACEIs or ARBs may demonstrate a good tolerability profile without adverse events in simple hypertensive diabetic patients [12]. Thus, though not recommended given in combination with ACEIs or ARBs, aliskiren was suggested to be used alone as an equivalent alternative to ACEIs or ARBs in type 2 DM patients with renal impairment [13]. However, it was not known if aliskiren, with both angiotensin II dependent and independent mechanisms, may prevent vascular impairment in the presence of DM.

Interestingly, aliskiren is recently shown to increase circulating EPC numbers in DM patients with hypertension [14]. In addition, the Vascular endothelial growth factor (VEGF) and chemokine stromal cell-derived factor-1α (SDF-1α) are reported to enhance the mobilization of bone marrow-derived EPCs and considered as markers of neovascularization [15,16]. Therefore, it raises a possibility that aliskiren may increase EPC numbers and prevent diabetic vasculopathy via the VEGF and SDF-1 related mechanisms. Thus, in this study, we sought to investigate whether and how aliskiren modifies EPC numbers and improves neovasculogenesis using DM animals with hindlimb ischemia, which is a model of diabetic peripheral vascular disease.

## Materials and Methods

### Diabetic animal model

The 6-week-old male FVB/NJNarl mice were purchased from the National Laboratory Animal Center (Taipei, Taiwan). The animals were raised according to the regulations of the Animal Care Committee of National Yang-Ming University. After a 2-week stabilization period, hyperglycemia was generated in 6-week-old male FVB/NJNarl mice by the intraperitoneal injection of streptozotocin (40 mg/kg for 5 days). In each animal, blood pressure and heart rate were measured regularly through a tail cuff using a noninvasive automatic sphygmomanometer. Body weight and blood sugar concentration were also monitored both at baseline and regularly. Overall, the mice should show a blood glucose level of at least 225 mg/dl during the second week before they could proceed to the following protocol.

### Experiment protocols

About 1 week after the diabetes was established with the confirmation of significantly increased blood glucose level, either aliskiren (5 or 25 mg/kg/day) diluted in PBS (n = 12 in each group), olmesartan (10 mg/kg/day) (n = 6), candesartan (20 mg/kg/day) (n = 6) or PBS alone (n = 12) were administered to the diabetic mice via an osmotic minipump (Alzet model 2006) for 6 weeks. Hydralazine (2 or 10 mg/kg/day) (n = 12 in each group) was given in the drinking water. In the hydralazine-treated group, we measured the amount of the water they drank daily. The blood pressures were also measured weekly to make sure the accurate drug delivery. The doses of the drugs were chosen according to the reference [10,17–20]. Aliskiren and olmesartan were kindly given from Novartis and Pfizer respectively. Candesartan and hydralazine were purchesed from Sigma.

**Fig 1:**
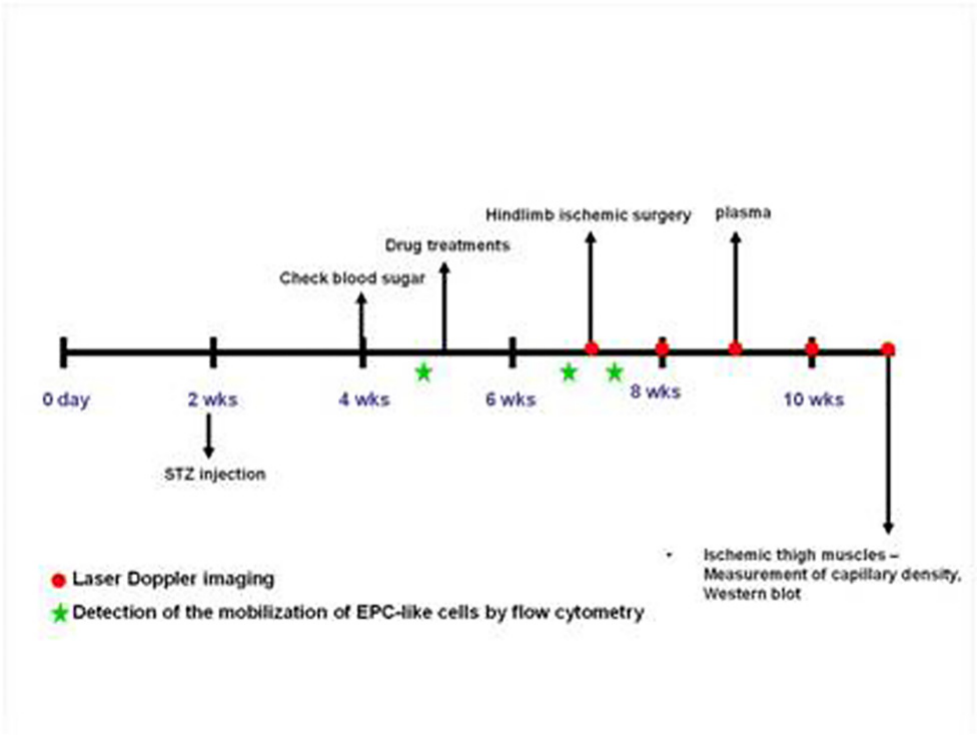
Time line of *in vivo* study.

Some diabetic mice received both aliskiren (25 mg/kg/day) and intraperitoneal injection of anti-SDF-1 neutralizing monoclonal antibody (mAb, 50 μg; R&D) 3 times per week up to 2 weeks. Mouse IgG1 isotype was administrated as a control. The protocol of animal study was approved by the Institutional Animal Care and Use Committee (IACUC) of National Yang-Ming University, Taipei, Taiwan, ROC. The study was conducted according to European Commission guidelines. **(Fig 1).**

### Mouse hindlimb ischemic model

After receiving different drug treatment for 2 weeks, the animals in the individual group were anaesthetized by i.p. injection of Xylocaine (2 mg/kg) plus Zoletil (5 mg/kg). The unilateral hindlimb ischemia was induced at the second week of the drug-treatment period by excising the right femoral artery. Briefly, the proximal and distal portions of the right femoral artery and the distal portion of the right saphenous artery were ligated. Hindlimb blood perfusion was measured regularly with a laser Doppler perfusion imager system (Moor Instruments Limited, Devon, UK). To avoid the influence of ambient temperature and the blood pressure changes of the animals, the results were expressed as the ratio of perfusion in the ischemic (index) versus nonischemic limb (control). The animals were sacrificed for the following studies 4 weeks after the establishment of hindlimb ischemia.

### Measurement of capillary density in the ischemic limb

Both the index and control limbs were fixed in 4%paraformaldehyde then embedded in paraffin. Sections were de-paraffinized and incubated with a rat-monoclonal antibody against murine CD31 (BD). Antibody distribution was visualized with the avidin-biotin-complex technique and Vector Red chromogenic substrate (Vector Laboratories), followed by counterstaining with hematoxylin. Three cross-sections were analyzed for each animal and ten different fields from each tissue preparation were randomly selected, and visible capillaries were counted.

### Detection of the mobilization of EPC-like cells by flow cytometry

The mononuclear cells were incubated with fluorescein isothiocyanate (FITC) anti-mouse Sca-1 (eBioscience) and phycoerythrin anti-mouse Flk-1 (VEGFR-2, eBioscience) antibodies at 4°C for 30 minutes. The expression of Sca-1^+^/ Flk-1^+^ cells (EPC-like cells) in mononuclear cells were analyzed by flow cytometry with a Cytomic FC 500 (Beckman-Coulter, Miami, FL). For analysis, 10^5^ circulating cells were quantified by enumerating Sca-1^+^/ Flk-1^+^ cells and were scored using a CXP software (Beckman-Coulter).

### Western blot of ischemic thigh muscles

Ischemic thigh muscle samples were prepared by homogenizer (MICCRAD9, ART Prozess & Labortechnik GmbH & Co. KG) on ice to avoid protein damage. Equal amounts of protein were subjected to SDS-PAGE electrophoresis using 4–12% gradient gels under reducing conditions (Bio-Rad Laboratories) and transferred to nitrocellulose membranes (GE Healthcare). Membranes were incubated with antibodies against Hypoxia-Inducible Factor (HIF)-1α (Novus), p-eNOS (Ser1177) (Millipore), VEGF (sc-507, Santa Cruz Biotechnology), SDF-1 (3530, Cell Signaling), and (P)RR (Novus). The immunoblotting expression of HIF-1α, VEGF, and SDF-1 was normalized using a mouse monoclonal anti-α-actin antibody.

### Quantitative RT-PCR of ischemic thigh muscles

HIF-1α, VEGF, SDF-1, and eNOS mRNA expression in mice ischemic thigh muscles were verified by quantitative RT-PCR. Total RNA was isolated using GeneJET RNA Purification Kit (Thermo Scientific) according to the manufacturer’s protocol. The complement DNA was synthesized from RNA using Maxima First Strand cDNA Synthesis Kit for RT-qPCR (Thermo Scientific). Mouse HIF-1α forward and reverse primers were: 5- AACCTGGCAATGTCTCCT-3 and 5- GCAACCTCTTGATTCAGTGCAG-3, respectively. Mouse VEGFA forward and reverse primers were: 5- TTTCGGGAACCAGACCTCTCA-3 and 5- AGGACTGTTCTGTCAAC-3, respectively. Mouse SDF-1 forward and reverse primers were: 5-CTCTGCATCAGTGACGGTAA-3 and 5-TTCAGCCGTGCAACAATC-3, respectively. Mouse eNOS forward and reverse primers were: 5-TATTTGATGCTCGGGACTGC-3 and 5-GGAACACTGTGATGGCT-3, respectively. Primers were purchased from GE Healthcare Dharmacon. The GAPDH served as an internal control. The assay was performed using the StepOnePlus Real-Time PCR System utilizing TaqMan Universal PCR mix (Applied Biosystems). Relative quantification determines the change in expression of target transcripts in the thigh muscles from treated mice relative to the thigh muscles from untreated diabetic mice (vehicle (PBS)-treated mice. Relative quantification was calculated using Applied Biosystems SDS software based on the equation RQ = 2^-ΔΔCt^, where RQ is relative quantification and Ct is the threshold cycle to detect fluorescence. Threshold cycle data were normalized to the internal standard, GAPDH, using the formula: ΔCt = target Ct–GAPDH Ct. Also, ΔΔCt was calculated as ΔΔCt = ΔCt (treated mice)– ΔCt (control). The primers and TaqMan probes for detection of HIF-1α, VEGF, SDF-1, eNOS, and GAPDH transcripts were from Applied Biosystems. In this part, we also used beta actin as the other housekeeping gene (Actin beta TaqMan Gene Expression Assay (ID: Mm00607939_s1, Life technologies, USA)) and conducted the same quantitative RT-PCR experiments.

### ELISA

Plasma concentrations of VEGF, SDF-1α, and angiotensin II proteins in mice at 14 days after hindlimb ischemia were determined by ELISA (mouse CXCL12/SDF-1α, MCX120, and VEGF ELISA kit, R&D system; angiotensin II SPIE-IA kit, Bertin Pharma) according to manufacturer’s instruction.

### Statistical analysis

Results are given as means ± standard errors of the mean (SEM). Statistical analysis was done by unpaired Student’s t test or analysis of variance, followed by Scheffe’s multiple-comparison *post hoc* test. SPSS software (version 14; SPSS, Chicago, IL, USA) was used to analyze data. A p value of <0.05 was considered statistically significant.

## Results

### Aliskiren improved neovasculogenesis and enhanced EPC-like cell mobilization in ischemic hindlimbs of diabetic mice

**Fig 2:**
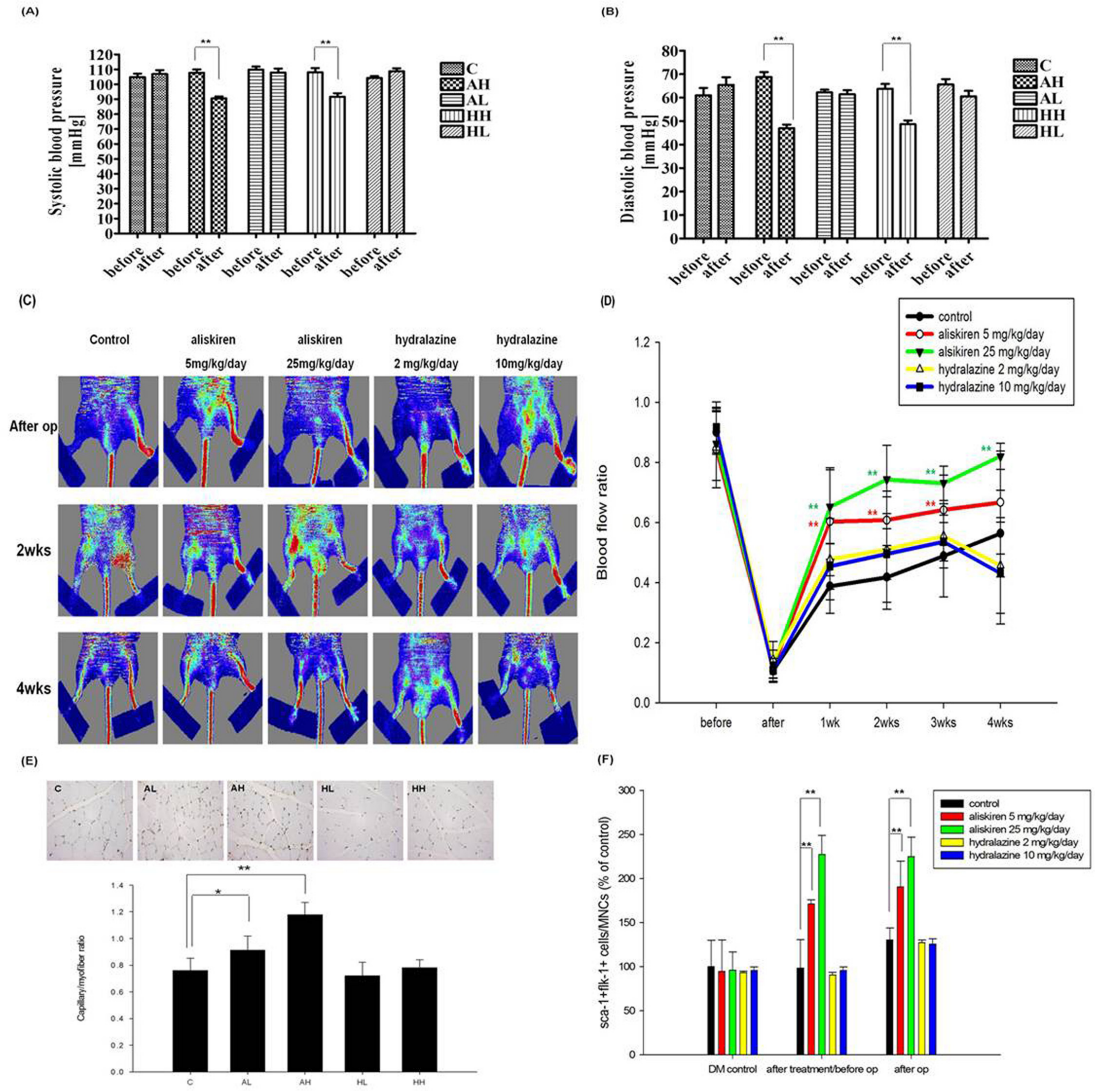
Aliskiren does-dependently improved neovasculogenesis and enhanced EPC-like cell mobilization in ischemic hindlimbs of diabetic mice. Systolic blood pressure (n = 12; A), diastolic blood pressure (n = 12; B). Foot blood flow monitored *in vivo* by LDI in each group of diabetic mice. Representative evaluation of the ischemic (right) and non-ischemic (left) hindlimbs, before, immediately after, and 4 weeks after the surgery. In color-coded images, red indicates normal perfusion and blue indicates a marked reduction in blood flow in the ischemic hindlimb. Blood flow recovery was markedly improved in aliskiren (5 or 25 mg/kg/day) treated mice compared with untreated mice (n = 12; C, D). Anti-CD31 immunostaining showed increased number of capillary formation in aliskiren (5 or 25 mg/kg/day) treated groups (E). EPC-like cell mobilization after tissue ischemia was determined by flow cytometry. Pretreatment with aliskiren (5 or 25 mg/kg/day) significantly enhanced the number of circulating EPC after ischemia (n = 6; F). C represented untreated diabetic mice (vehicle (PBS)-treated mice); AL represented aliskiren low dose (5 mg/kg/day); AH represented aliskiren high dose (25 mg/kg/day); HL represented hydralazine low dose (2 mg/kg/day); HH represented hydralazine high dose (10 mg/kg/day). Statistical analysis was done by unpaired Student’s t test or analysis of variance, followed by Scheffe’s multiple-comparison *post hoc* test. A p value of <0.05 was considered statistically significant. *p < 0.05, **p < 0.01 *c*ompared with same group before treatment.

The animals treated with high dose and low dose of hydralazine were used as blood pressure control groups. There were no differences in blood pressure reduction between aliskiren treated group and hydralazine treated group either in high dose comparison or in low dose comparison **(Fig 2A and 2B)**. Blood flow in the ischemic hindlimb was equally reduced by hindlimb ischemia surgery in each group of diabetic mice **(Fig 2C).** Perfusion recovery was markedly attenuated in the hydralazine or PBS groups compared with the aliskiren groups during the postoperative weeks **(Fig 2C and 2D)**. Immunohistochemical analysis revealed that capillary density in the ischemic limb was increased in the aliskiren treated mice compared with that in PBS treated mice. There was no significant increase in the hydralazine treatment group than in PBS treated group **(Fig 2E)**. Furthermore, the number of EPC-like cells was increased in a dose-dependent manner by aliskiren treatment (5 or 25 mg/kg/day) for 2 weeks in diabetic mice. The similar increase was also seen after the induction of hindlimb ischemia **(Fig 2F).** Furthermore, immunostaining data revealed that aliskiren could increase both CD34-positive homed hematopoietic stem precursor cells and CXCR4 signals at the ischemic site of diabetic mice **(S1 Fig).** These findings indicated that aliskiren could enhance neovasculogenesis in response to tissue ischemia in a dose-dependent manner in diabetic mice. The beneficial effects of aliskiren could be independent to blood pressure reduction since blood pressure changes were similar between aliskiren and hydralazine treatment groups **(Fig 2A and 2B)**.

### Aliskiren up-regulated muscular HIF-1α, VEGF, SDF-1α, and (P)RR expression in ischemic limbs

**Fig 3:**
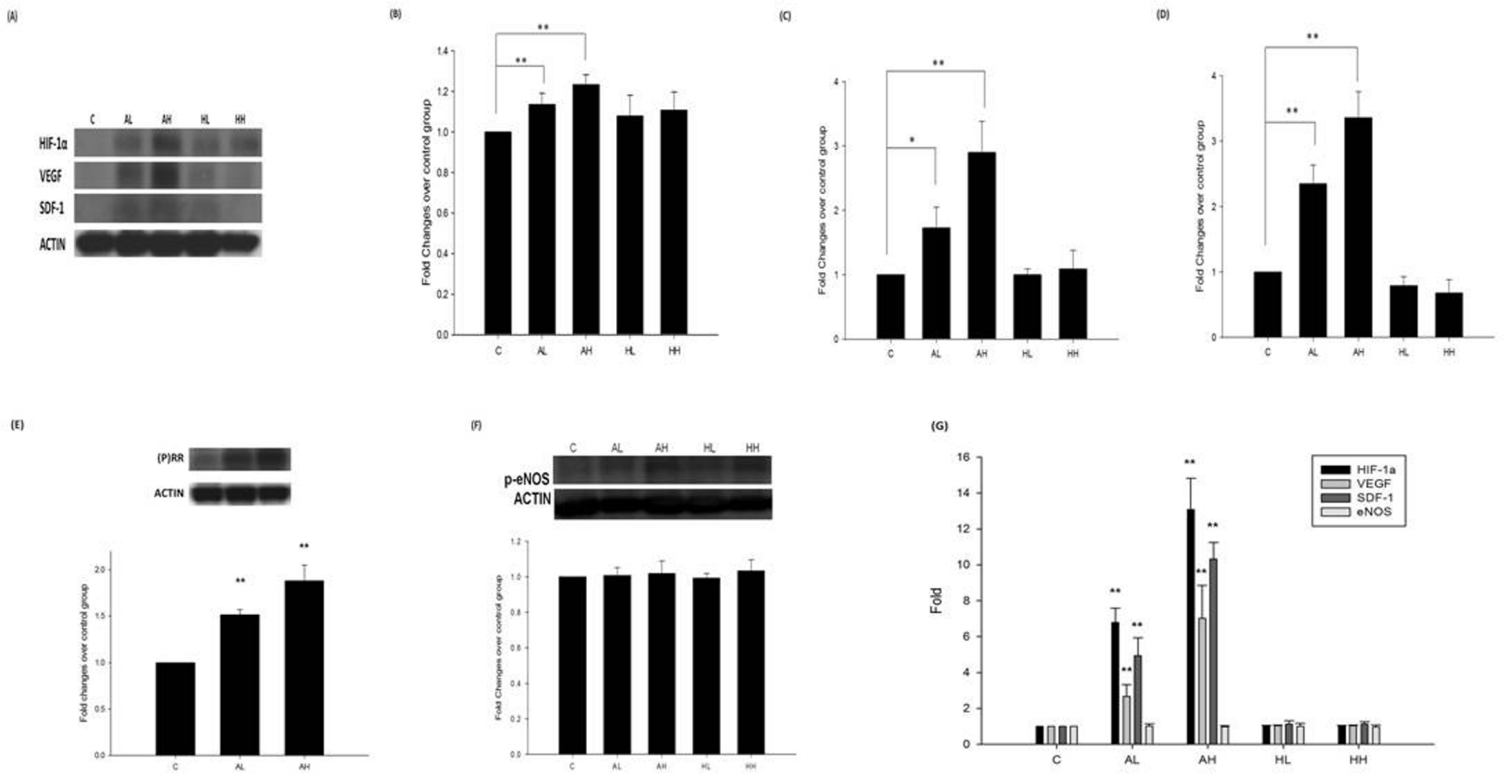
Differential effects of aliskiren and hydralazine on protein and mRNA expressions in index thigh muscles in diabetic mice with hindlimb ischemia. Western blots of HIF-1, VEGF, SDF-1α, and actin (n = 6; A). The individual expression of HIF-1, VEGF and SDF-1α was increased in aliskiren (5 or 25 mg/kg/day) treated group (n = 6; B, C, D). (P)RR expressions were also enhanced in aliskiren-treated groups (n = 6; E). However, the expression of p-eNOS was not changed by aliskiren (n = 6; F). Quantitative RT-PCR for the effects of aliskiren on mRNA expressions in thigh muscles in diabetic mice with hindlimb ischemia (n = 6; G). N represents thigh muscles from n different individuals, and thigh muscles from each individual were experimented for 3 independent experiments. C represents untreated diabetic mice (vehicle (PBS)-treated mice); AL represents aliskiren in low dose (5 mg/kg/day); AH represents aliskiren in high dose (25 mg/kg/day); HL represents hydralazine in low dose (2 mg/kg/day); HH represents hydralazine in high dose (10 mg/kg/day). Statistical analysis was done by unpaired Student’s t test or analysis of variance, followed by Scheffe’s multiple-comparison *post hoc* test. A p value of <0.05 was considered statistically significant. *p < 0.05, **p < 0.01 compared with untreated diabetic mice (vehicle (PBS)-treated mice).

Compared with PBS treatment, aliskiren (5 or 25 mg/kg/day) but not hydralazine (2 or 10 mg/kg/day) dose-dependently increased the expression of HIF-1α **(Fig 3B)** VEGF **(Fig 3C)**, or SDF-1α **(Fig 3D).** The co-immunoprecipitation analysis also showed that SDF-1 did complex with CXCR4 **(S2 Fig)**. Aliskiren also enhanced (P)RR expression in ischemic limbs compared with untreated diabetic mice **(Fig 3E)**. Neither aliskiren nor hydralazine altered the expression of phosphorylated eNOS (p-eNOS) **(Fig 3F).** The plasma concentration of nitrite/nitrate was not changed after aliskiren or hydralazine treatments **(S3 Fig)**. We also used L-NAME, an NO inhibitor, to teste whether NO was involved in the aliskiren related mechanism by co-treatment with aliskiren and L-NAME. The results showed that co-treatment with L-NAME do not abolish the beneficial effects of aliskiren on decreased blood pressures and on angiogenesis **(S4 Fig)**. The effects of aliskiren on mRNA expressions in thigh muscles in diabetic mice with hindlimb ischemia were also confirmed by quantitative RT-PCR. Aiskiren dose-dependently increased the mRNA expression of HIF-1α, VEGF, and SDF-1α while GAPDH was used as the housekeeping gene **(Fig 3G).** The similar results were also shown with beta actin as the other housekeeping gene **(S5 Fig)**.

### Aliskiren increased plasma angiogenic cytokine SDF-1α and VEGF levels after hindlimb ischemia

**Fig 4:**
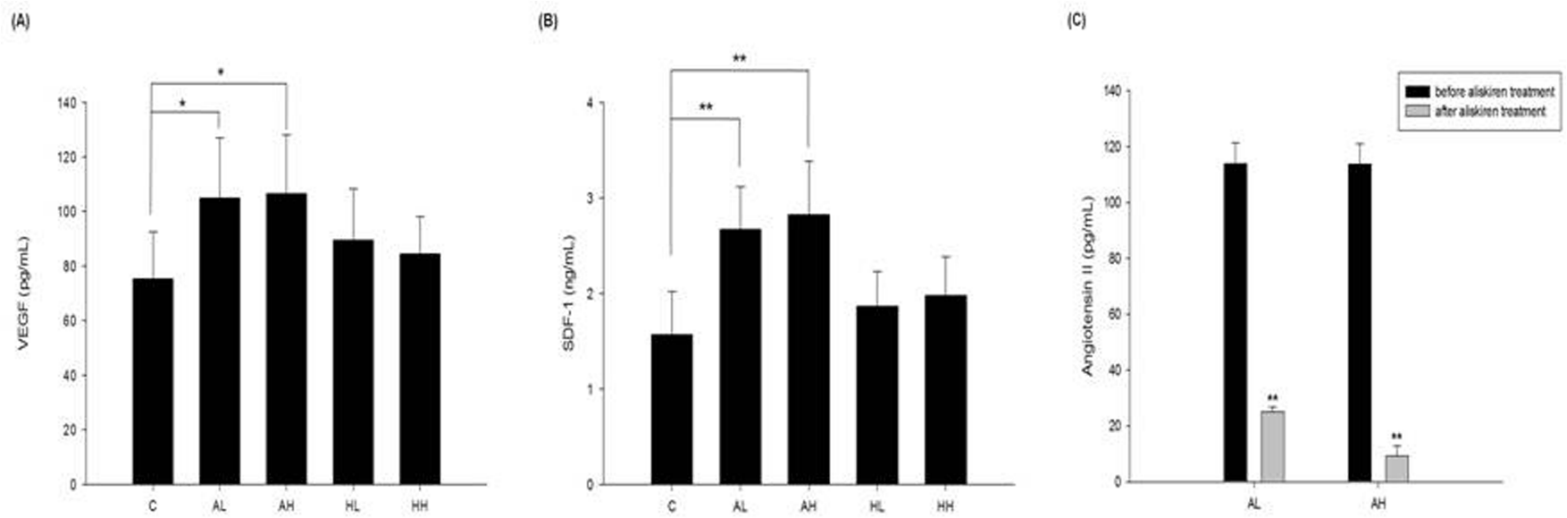
Differential effects of aliskiren and hydralazine on protein expressions in plasma in diabetic mice with hindlimb ischemia. The individual plasma level of VEGF and SDF-1α was increased in aliskiren (5 or 25mg/kg/ day) treated groups (n = 6; A, B). Angiotensin II concentrations were decreased after aliskiren treatments in ischemic mouse model of diabetic mice (n = 6; C). C represents untreated diabetic mice (vehicle (PBS)-treated mice); AL represents aliskiren in low dose (5 mg/kg/day); AH represents aliskiren in high dose (25 mg/kg/day); HL represents hydralazine in low dose (2 mg/kg/day); HH represents hydralazine in high dose (10 mg/kg/day). Statistical analysis was done by unpaired Student’s t test or analysis of variance, followed by Scheffe’s multiple-comparison *post hoc* test. A p value of <0.05 was considered statistically significant. *p < 0.05, **p < 0.01 compared with untreated diabetic mice (vehicle (PBS)-treated mice).

ELISA assay was performed on mice plasma 2 weeks after ischemic surgery. Plasma SDF-1α and VEGF levels were significantly increased by aliskiren (5 or 25 mg/kg/day) but not by hydralazine (2 or 10 mg/kg/day) treatment in comparison with that by PBS **(Fig 4A and 4B).** Besides, angiotensin II concentrations were decreased after aliskiren treatments in ischemic mouse model of diabetic mice **(Fig 4C)**.

### SDF-1α was essential to the effects of aliskiren on neovasculogenesis after hindlimb ischemia

**Fig 5:**
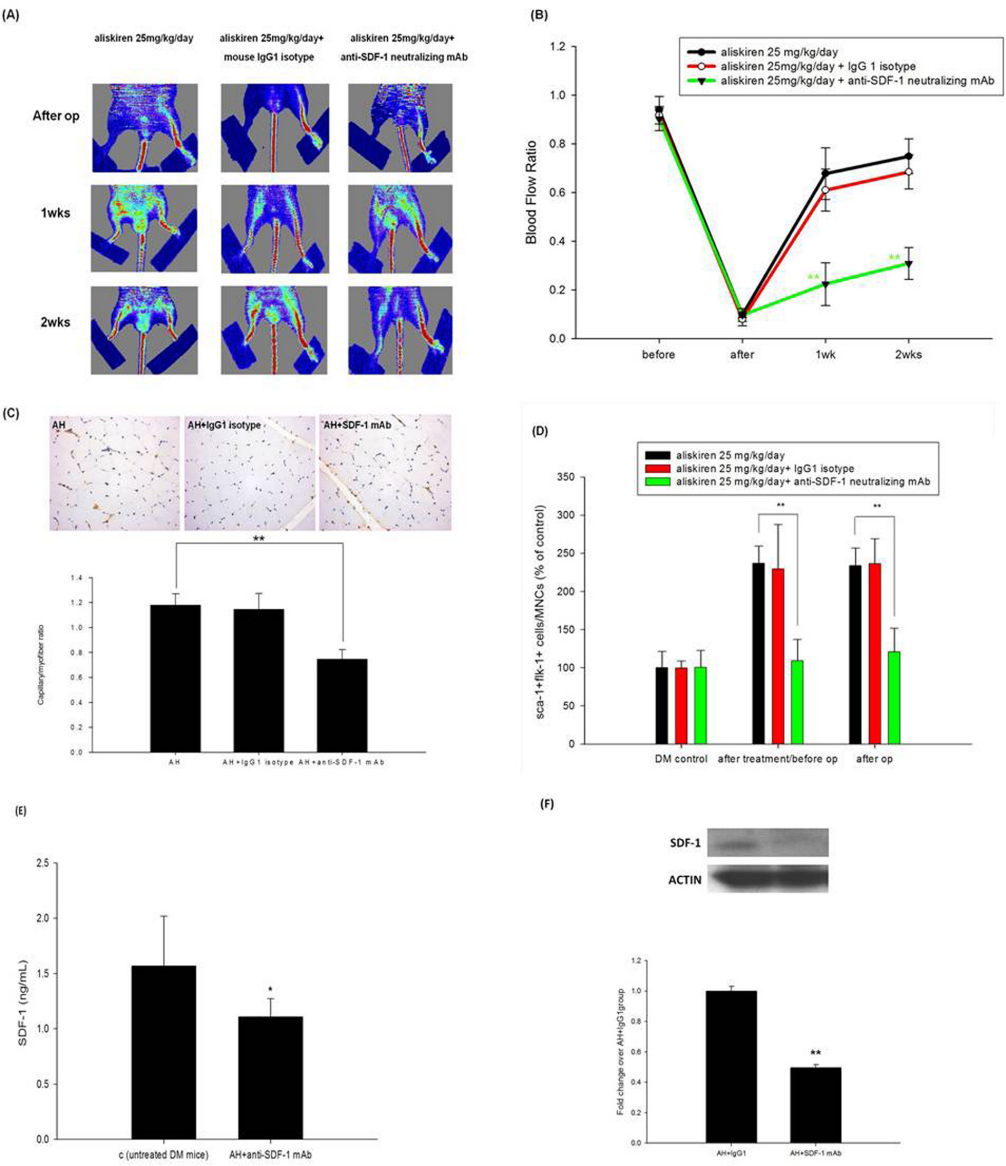
SDF-1α dependent effects of aliskiren on neovasculogenesis after hindlimb ischemia. In color-coded images, red indicates normal perfusion and blue indicates a marked reduction in blood flow in the ischemic hindlimb. Quantitative analysis of the LDI ratio for foot blood flow in animals before, immediately after, and 2 weeks after the surgery of hindlimb ischemia. Compared with aliskiren (25 mg/kg/day) treatment alone, aliskiren (25 mg/kg/day) in combination of anti-SDF-1 mAb treatment significantly impaired blood flow recovery after hindlimb ischemia (n = 6 in each group; A, B). Anti-CD31 immunostaining showed decreased number of capillary formation in the animals with both aliskiren and anti-SDF-1 mAb treatment as compared with that with aliskiren treatment alone (n = 6; C). Ischemia-induced EPC-like cell mobilization was determined by flow cytometry. Compared with aliskiren treatment alone, the combination of aliskiren and anti-SDF-1 mAb treatment significantly attenuated the number of circulating EPCs (n = 6; D). ELISA data showed that the amount of SDF-1 in plasma of aliskiren high dose (25 mg/kg/day) with anti-SDF-1 neutralizing antibody was significantly lower than control (untreated DM mice; vehicle (PBS)-treated mice) (n = 6; E). SDF-1 protein was abolished in index thigh muscles by the neutralizing antibody after injection of anti-SDF-1 neutralizing antibody for 2 weeks (n = 6; F). Statistical analysis was done by unpaired Student’s t test or analysis of variance, followed by Scheffe’s multiple-comparison *post hoc* test. A p value of <0.05 was considered statistically significant. *p < 0.05, **p < 0.01.

Intraperitoneal injection of the anti-SDF-1 neutralizing mAb abolished the effects of aliskiren (25 mg/kg/day) **(Fig 5A and 5B).** Compared with aliskiren alone, the combined treatment with aliskiren and anti-SDF-1 mAb significantly reduced the capillary density in the ischemic limb **(Fig 5C)** and the number of circulating EPC-like cells either before or after hindlimb ischemia surgery **(Fig 5D).** Also, anti-SDF-1 neutralizing mAb impaired endogenous SDF-1 in plasma and ischemic muscles of diabetic mice **(Fig 5E and 5F)**. In the *in vitro* study, AT1 and AT2 siRNA did not abolished the effects of aliskiren (10 μM) on the expression of SDF-1 on EPCs from diabetic mice **(S6 Fig)**.

### Co-treatment of aliskiren and ARBs did not intensify neovasculogenesis after hindlimb ischemia

**Fig 6:**
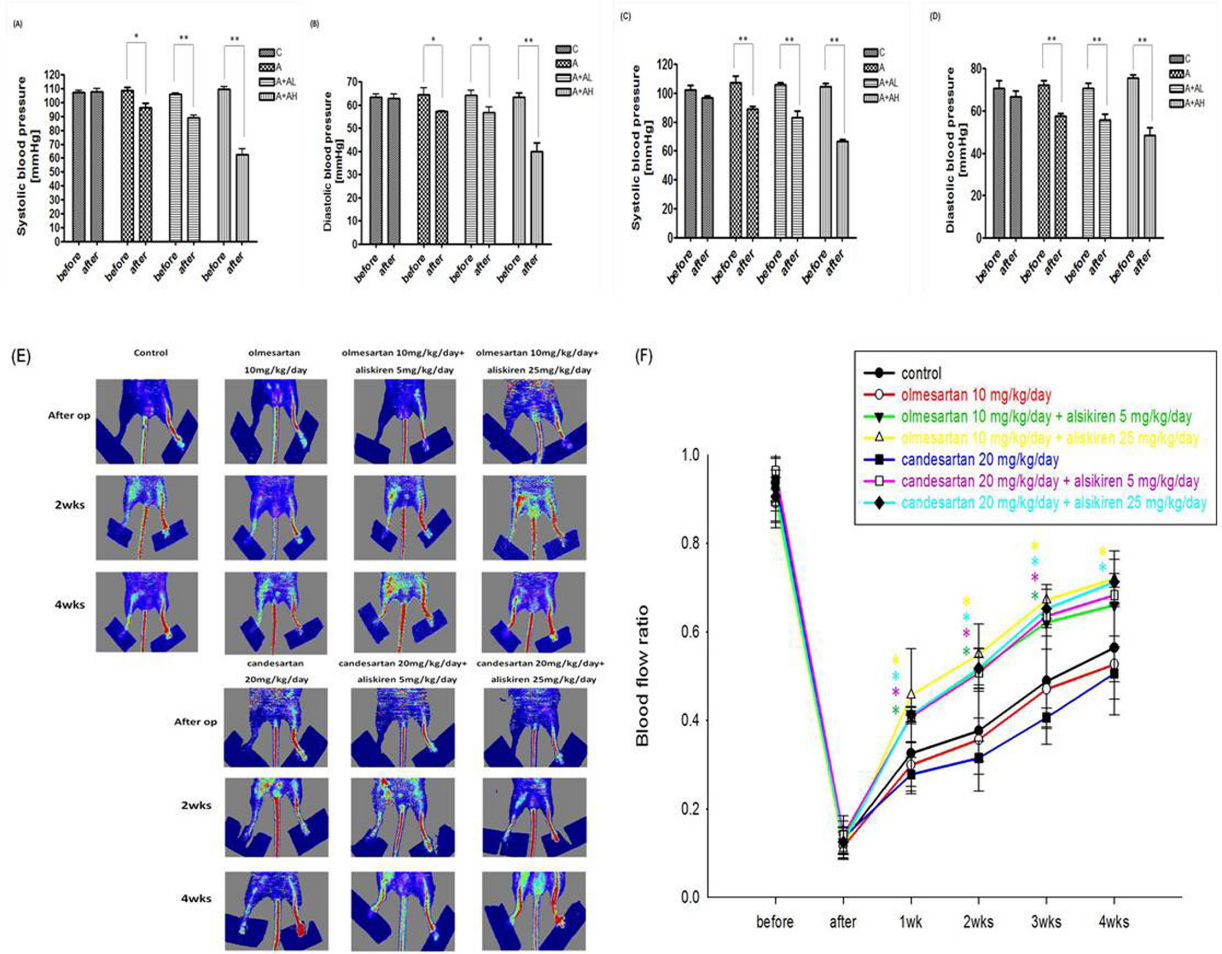
Angiotensin II independent effects of aliskiren on neovasculogenesis after hindlimb ischemia. Effects of ARBs such as olmesartan or candesartan and combination treatment of ARBs and aliskiren on blood pressure *in vivo*. Olmesartan, 10 mg/kg/day (n = 6; A, B); candesartan, 20 mg/kg/day (n = 6; C, D). Color-coded and quantitative analysis of the LDI ratio for foot blood flow in animals of hindlimb ischemia (n = 6; E, F). Compared with control group, the combination of aliskiren with ARBs treatment, but not ARBs alone, enhanced blood flow recovery after hindlimb ischemia. Thus, there seems no additional effect with ARBs to aliskiren on the recovery of blood flow. A+AL represented combination of olmesartan or candesartan and aliskiren low dose (5 mg/kg/day); A+AH represented combination of olmesartan or candesartan and aliskiren high dose (25 mg/kg/day). Statistical analysis was done by unpaired Student’s t test or analysis of variance, followed by Scheffe’s multiple-comparison *post hoc* test. A p value of <0.05 was considered statistically significant. *p < 0.05, **p < 0.01.

Blood pressures in all groups were monitored during the experimental period **(Fig 6A, 6B, 6C and 6D)**. Treatment with ARBs such as olmesartan (10 mg/kg/day) or candesartan (20 mg/kg/day) alone did not improve blood flow recovery after hindlimb ischemia compared to that of the control group, suggesting there was no significant effect of ARBs on the recovery of blood flow after hindlimb ischemia in this diabetic animal model. On the other hand, aliskiren (5 or 25 mg/kg/day) co-treated with olmesartan (10 mg/kg/day) or candesartan (20 mg/kg/day) significantly increased blood flow recovery **(Fig 6E and 6F)**. However, the effects of these co-treated groups were similar to that of the aliskiren (5 or 25mg/kg/day) alone groups **(Fig 2C and 2D)**, suggesting there was no additional effect with ARBs to aliskiren on the recovery of blood flow. Besides, the beneficial effects of aliskiren could be independent to blood pressure since the blood pressure reduction levels in aliskiren 25 mg/kg/day group were in similar extent with blood pressure reduction levels in olmesartan 10 mg/kg/day group or candesartan 20 mg/kg/day **(Fig 6A, 6B, 6C and 6D)**.

## Discussion

In this study, we have uncovered new insights of aliskiren in ischemia-induced neovasculogenesis in diabetic mice. First, treatment of aliskiren facilitated the recovery of limb perfusion and capillary density, in addition to increasing the number of circulating Sca-1^+^/Flk-1^+^ EPC-like cells and elevating the expression level of the VEGF, SDF-1α, and (P)RR. Second, the *in vivo* effects of aliskiren depended on the SDF-1 rather than the alternation of blood pressure. Third, classical angiotensin II pathways may not participate in ischemic-induced angiogensis and *in vivo* neovacularization in the presence of aliskiren. Taken together, this study provided the first evidence that aliskiren enhanced ischemia-induced neovasculogenesis in a dose-dependent manner via VEGF/SDF-1α related mechanisms in diabetic mice.

Previous studies suggested that VEGF, SDF-1α, and circulating EPC number could be markers of neovascularization [21–23]. Recently, aliskiren was shown to enhance EPC number in ApoE-/- mice [10]. However, it was also reported to reduce the EPC number in patients with early atherosclerosis [24]. Given that the previous data were contradictory, our results were the first to demonstrate that aliskiren could not only increase EPC-like cell numbers, but it also has beneficial effects on ischemic-induced neovascularization in diabetic animals. Both might occur in parallel.

HIF-1α is a critical transcription factor involved in oxygen homeostasis and regulating various adaptive responses to hypoxia in neovascularization [25]. Moreover, superoxide induced by hyperglycemia may destabilize HIF-1α and reduce the activation of SDF-1α, VEGF and CXCR4 in response to hypoxia [26,27]. In this study, aliskiren was shown to enhance HIF-1α, VEGF and SDF-1α expression in ischemic limbs of type I diabetic animals with high blood glucose. Furthermore, western blot assays showed that aliskiren (5 or 25 mg/kg/day) treatment increased the expression of both (P)RR and HIF-1 alpha in ischemic thigh muscles. Taken together, aliskiren treatments were accompanied with the enhancements of (P)RR and HIF-1 alpha in the ischemic thigh muscles of diabetic mice although previous studies showed that (P)RR, a vacuolar H⁺-ATPase [28], was increased in the hearts of diabetic rats and decreased with aliskiren treatments [29]. However, the direct effects of (P)RR on HIF-1 alpha still need further investigations. Also, the interaction between (P)RR and aliskiren still needs further evidence because previous study showed that aliskiren did not affect (pro)renin binding to its receptors [30]. Though the detailed molecular mechanisms are not completely elucidated, aliskiren was demonstrated to activate HIF-1α-VEGF-SDF-1α related pathways and enhance neovascularization in this diabetic animal model.

Accordingly, in mice with hindlimb ischemia, VEGF-mediated neovascularization may partially depend on the activation of SDF-1α-CXCR4 pathways [31,32]. In our study, aliskiren increased the expression of VEGF and SDF-1α. In addition, aliskiren modulated *in vivo* vasculogenesis that could be significantly suppressed by intra-peritoneal injection of an anti-SDF-1 neutralizing mAb, suggesting the critical role of SDF-1α in aliskiren-induced neovascularization. However, there were a number of issues that still need to be further investigated in our current model, including 1) the number of CXCR4^+^ cells under the treatment of aliskiren; 2) the effect of anti-VEGF neutralizing mAb and 3) the local recruitment of EPC-like cells to the site of neoangiogenesis/ integration into the neovessels.

Recent study suggested that renin inhibition modulated angiogenesis via affecting the angiopoietin-1/angiopoietin-2 ratio independent of blood pressure and resulted in higher capillary density in spontaneously hypertensive rats [33]. In the current study, the roles of aliskiren on neovascularization were also independently of blood pressure in another different diabetic animal model. On the other hand, aliskiren was previously suggested to provide its vascular protection effects via the activation of eNOS, the increase in NO bioavailability, and the enhancement of anti-inflammatory effects in either the mechanically-injured artery model or the atherosclerosis model [9]. Aliskiren could also protect against myocardial I/R injury through activation of the PI3K-Akt-eNOS pathway [34]. However, in our diabetic animal model, which is different from previous ones, aliskiren did not alter eNOS expression in ischemic limbs. Furthermore, the plasma concentrations of nitrite/nitrate were not changed by aliskiren and the effects of aliskiren on neovasculogenesis were not abolished by L-NAME in DM mice, suggesting the functions of aliskiren in neovascularization are eNOS-independent.

Theoretically, the beneficial effects of aliskiren could be related to its function on angiotensin II or (P)RR pathways or both. While some ARBs were suggested to increase basal EPC number and function *in vivo* [35–37], ARB alone has not been shown to improve but even block the *in vivo* neovascularization [38] since angiotensin II may be required for neovascularization especially under stimulation [39]. In the current study, ARB such as olmesartan or candesartan, in a potentially maximal dose [19,40], did not improve the ischemic-related neovascularization in diabetic animals. Although RAS blockade seems to play a controversial role on neovascularization [20, 39, 41], our findings are in concordance to the previous studies that ARB such as candesartan exerts no benefits to neovascularization in diabetic animals. Candesartan was shown to inhibit neovascularization in both type 1 diabetic mice and Otsuka Long-Evans Tokushima Fatty rats, a model of human non-insulin-dependent DM [20,42] and had no effects on circulating angiogenic factor VEGF level in patients with acute myocardial infarction [43]. Taken together, the blockage on angiotensin II pathways might not improve *in vivo* neovascularization in disease conditions such as DM. Our findings could be further supported by the previous findings that the angiotensin II pathways did not contribute to the pro-neovascularization effects of ACEI in diabetic animals [20].

The effects of aliskiren on neovascularization in diabetic-related status might be independent of the classic angiotensin II pathways. The independent beneficial effects of aliskiren could be supported by the recent findings that aliskiren on top of ARB may further improve cardiac neovascularization in a mouse model of myocardial infarction [11]. On the other hand, we could not completely exclude the effects of reduced angiotensin II level by aliskiren in our study. One of the possibilities might be due to the fact that ARBs would not have any effect anymore because of a low level of angiotensin II in the presence of aliskiren. Furthermore, the combination of aliskiren and ARB did not enhance the effects. As a matter of fact, the combination of aliskiren with ACEI/ARB therapy failed to reduce the progression of atheroma in patients with established cardiovascular disease [44]. Therefore, aliskiren seemed to have pleiotropic effects because ARB alone did not have any effect. Different from that of ACEI/ARBs, the novel mechanisms for the effects of aliskiren might provide another clue to the new potential therapeutic target in vascular repair and regeneration especially in the presence of Type II diabetes.

Aliskiren could selectively reduce *in vivo* (P)RR expression in glomeruli, renal tubules, and renal cortical vessels in diabetic rats [45]. In contrast, our results provided the first evidence that aliskiren could increase EPC number along with increased (P)RR expression in ischemic muscles of diabetic mice. These data implied that aliskiren may participate in the (P)RR signaling pathway. Since there is a lack of *in vivo* evidence that aliskiren could directly activate (P)RR, it is likely that the effects of aliskiren on the alternation of SDF and (P)RR expression are an epi-phenomenon. In addition, a previous study showed that the binding of renin with aliskiren had no effect on (P)RR signaling [46], suggesting that the renin-aliskiren complex may activate the (P)RR pathway through complicated mechanisms. To elucidate the possible structure and the detail action mechanism of aliskiren in *in vivo* diabetic models, further studies will thus be required.

Co-immunoprecipitation results revealed that the active SDF-1α binds with CXCR4 proteins in thigh muscle samples of the animals. Since the SDF is usually rapidly cleaved *in vivo*, these results suggested binding with the CXCR4 stabilized SDF-1α, resulting in continuous activation of the SDF-1α. These findings may as well support the notion that in the presence of aliskiren, the SDF-1α can bind to CXCR4 proteins and remain active in the thigh muscles of the ischemic limb of mice. Furthermore, given that intraperitoneal injection of the SDF-1 mAb could abolish the *in vivo* beneficial effects of aliskiren, it is possible that the activation of (P)RR and VEGF-SDF-1 pathways might contribute to the recovery of ischemic-induced neovascularization in the hindlimbs of diabetic animals. Although our data suggested that aliskiren treatment *in vivo* could enhance SDF-1 levels, we could not completely exclude the possibility of epi-phenomena. Moreover, even if we had used hydralazine to be a control group of the decreased blood pressure and followed their blood pressure during the experimental period, we still could not totally exclude the effects of blood pressure.

As we cannot conclude the effects of other ARBs, our findings did suggest the potential contribution of a novel (P)RR-VEGF-SDF1α signal pathway rather than the classic angiotensin II pathways to the beneficial effects of aliskiren on neovascularization especially in the high glucose or diabetic status.

## Conclusions

Aliskiren being a renin inhibitor, could not only increase circulating EPC numbers, but also improve the impaired neovascularization in diabetic mice. The therapeutic effects might be related to the stabilization of the HIF-1α and up-regulated VEGF/SDF-1α via the angiotensin II-independent pathways. Given the direct and independent vascular benefits of aliskiren, it is worthy to evaluate clinically the potential effects of aliskiren on blood pressure control and peripheral vascular diseases in diabetic hypertensives.

## Supporting Information

S1 Fig Effects of aliskiren on EPC homing and CXCR4 expression at ischemic sites in diabetic mice. Immunostaining of ischemic hind limb muscle with anti-CD34 antibody conjugated to Alexa Fluor 492 (green) and anti-CXCR4 antibody conjugated to Alexa Fluor 594 (red) in diabetic mice treated with aliskiren. The CD34-positive homed hematopoietic stem precursor cells were indicated with white arrows. Aliskiren treated group increased CD34/CXCR4-double-positive cells (arrow) in ischemic muscle compared with the control group. Hoechst dye (blue) was used to counterstain the nucleus. The ischemic hind limb tissue was evaluated by fluorescence microscopy at a magnification of 400x (Fig A in S1 Fig). The bar graph shows the CD34 positivity/myofiber ratio (Fig B in S1 Fig). H&E stainings of the muscle section (Fig C in S1 Fig). C represented untreated diabetic mice (vehicle (PBS)-treated mice); AL represented aliskiren low dose (5 mg/kg/day); AH represented aliskiren high dose (25 mg/kg/day). *p < 0.05, **p < 0.01 compared with untreated diabetic mice (vehicle (PBS)-treated mice).
(TIFF)

S2 Fig Co-immunoprecipitation analysis showed that SDF-1 did complex with CXCR4. Thigh muscle samples were from aliskiren 25 mg/kg/day treated group. Immunoprecipitation with antibody against CXCR4 and immunoblot analysis with antibody against SDF-1 or immunoprecipitation with antibody against SDF-1 and immunoblot analysis with antibodies against CXCR4.
(TIFF)

S3 Fig The plasma concentration of nitrite/nitrate. Total nitric oxide metabolites (nitrates plus nitrites) at 14 days after hindlimb ischemia were determined by Total Nitric Oxide and Nitrate/Nitrite Assay ELISA kit (KGE001, R&D system) according to manufacturer’s instruction. NOx represented the stable end product of NO. (n = 6 in each group).
(TIFF)

S4 Fig The effects of aliskiren on neovasculogenesis did not abolished by L-NAME in DM mice. Systolic blood pressure (n = 12 in AH group, n = 6 in AH+L-NAME group; Fig A in S4 Fig), diastolic blood pressure (n = 12 in AH group, n = 6 in AH+L-NAME group; Fig B in S4 Fig). Foot blood flow monitored *in vivo* by LDI in each group of diabetic mice. Blood flow recovery was markedly improved in either aliskiren (25 mg/kg/day) treated mice (n = 12; Fig C and D in S4 Fig) or aliskiren and L-NAME (30 mg/kg/day) co-treated group (n = 6; Fig C and D in S4 Fig). AH represented aliskiren high dose (25 mg/kg/day); AH+L-NAME represented aliskiren high dose combined with L-NAME (30 mg/kg/day). *p < 0.05, **p < 0.01 *c*ompared with same group before treatment.
(TIFF)

S5 Fig Aliskiren upregulated HIF-1α, VEGF, and SDF-1 mRNA in index thigh muscles in diabetic mice with hindlimb ischemia. Quantitative RT-PCR for the effects of aliskiren on mRNA expressions in thigh muscles in diabetic mice with hindlimb ischemia (n = 6). Threshold cycle data were normalized to the internal standard, beta actin. C represents untreated diabetic mice (vehicle (PBS)-treated mice); AL represents aliskiren in low dose (5 mg/kg/day); AH represents aliskiren in high dose (25 mg/kg/day); HL represents hydralazine in low dose (2 mg/kg/day); HH represents hydralazine in high dose (10 mg/kg/day). *p < 0.05, **p < 0.01 compared with untreated diabetic mice (vehicle (PBS)-treated mice).
(TIFF)

S6 Fig The enhanced SDF-1 expression by aliskiren was independent of AT1 and AT2 on EPCs from diabetic mice. Western blot and statistical analysis of AT1, AT2, and SDF-1 expressions after slencing with AT1 and AT2 siRNA (n = 6). AT1 and AT2 siRNA did not abolished the effects of aliskiren (10 μM) on the expression of SDF-1 on EPCs from diabetic mice. C represents untreated cells; siC represents control siRNA; siA represents co-treated AT1 and AT2 siRNA; siA+ali represents combined treatment of AT1 siRNA, AT2 siRNA, and aliskiren (10 μM).*p < 0.05, **p < 0.01 compared with untreated cells.
(TIFF)
