# Supplementary figures and images for: Direct Renin Inhibition with Aliskiren Improves Ischemia-Induced Neovasculogenesis in Diabetic Animals via the SDF-1 Related Mechanism
Source: PLoS One. 2015 Aug 25;10(8):e0136627. doi: 10.1371/journal.pone.0136627 (PMC4549314; doi:10.1371/journal.pone.0136627)

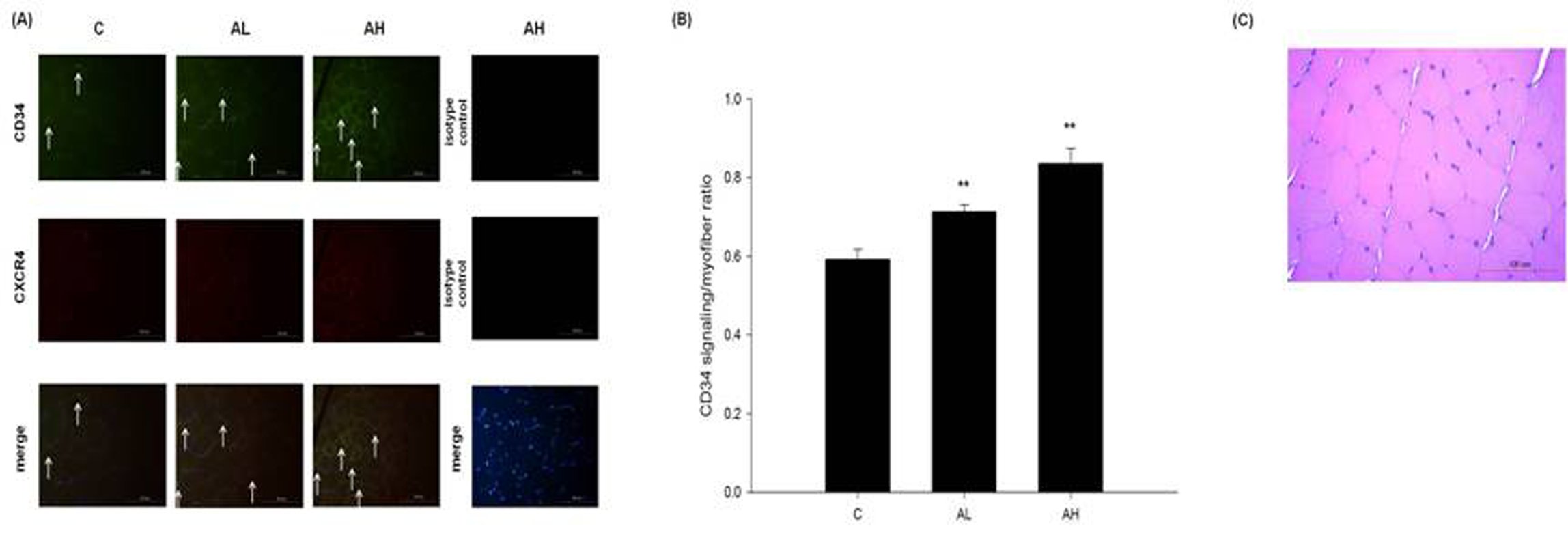

Supplement: S1 Fig — Immunostaining of ischemic hind limb muscle with anti-CD34 antibody conjugated to Alexa Fluor 492 (green) and anti-CXCR4 antibody conjugated to Alexa Fluor 594 (red) in diabetic mice treated with aliskiren. The CD34-positive homed hematopoietic stem precursor cells were indicated with white arrows. Aliskiren treated group increased CD34/CXCR4-double-positive cells (arrow) in ischemic muscle compared with the control group. Hoechst dye (blue) was used to counterstain the nucleus. The ischemic hind limb tissue was evaluated by fluorescence microscopy at a magnification of 400x (Fig A in S1 Fig). The bar graph shows the CD34 positivity/myofiber ratio (Fig B in S1 Fig). H&E stainings of the muscle section (Fig C in S1 Fig). C represented untreated diabetic mice (vehicle (PBS)-treated mice); AL represented aliskiren low dose (5 mg/kg/day); AH represented aliskiren high dose (25 mg/kg/day). *p < 0.05, **p < 0.01 compared with untreated diabetic mice (vehicle (PBS)-treated mice). (TIFF) [file pone.0136627.s001.tiff]

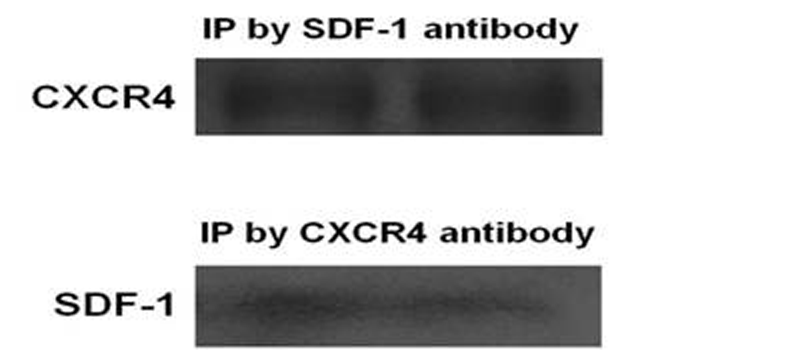

Supplement: S2 Fig — Thigh muscle samples were from aliskiren 25 mg/kg/day treated group. Immunoprecipitation with antibody against CXCR4 and immunoblot analysis with antibody against SDF-1 or immunoprecipitation with antibody against SDF-1 and immunoblot analysis with antibodies against CXCR4. (TIFF) [file pone.0136627.s002.tiff]

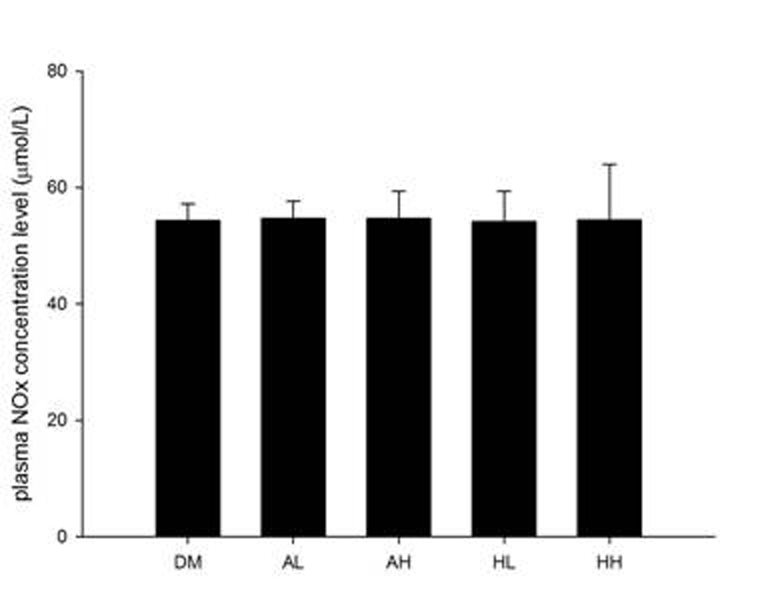

Supplement: S3 Fig — Total nitric oxide metabolites (nitrates plus nitrites) at 14 days after hindlimb ischemia were determined by Total Nitric Oxide and Nitrate/Nitrite Assay ELISA kit (KGE001, R&D system) according to manufacturer’s instruction. NOx represented the stable end product of NO. (n = 6 in each group). (TIFF) [file pone.0136627.s003.tiff]

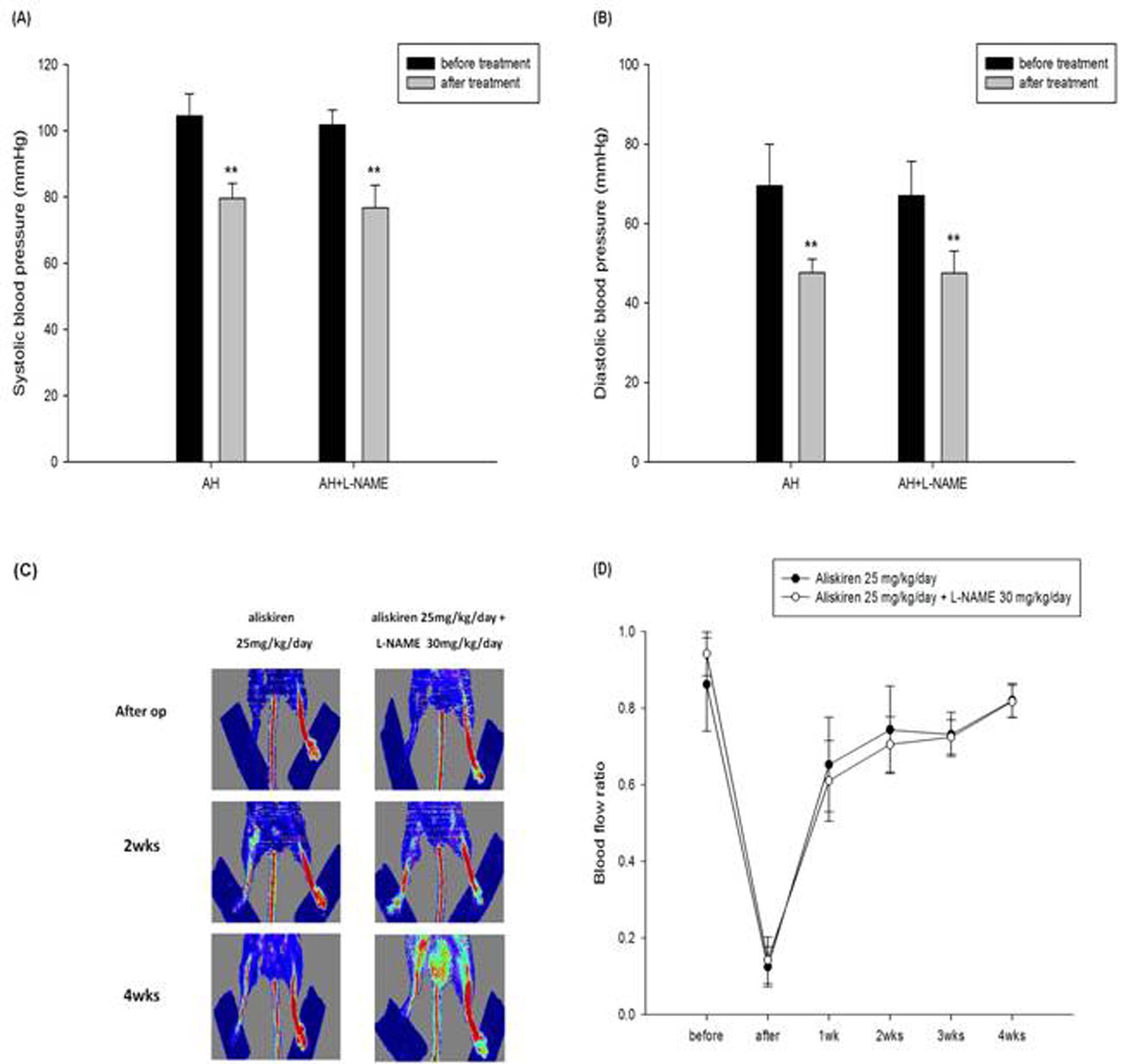

Supplement: S4 Fig — Systolic blood pressure (n = 12 in AH group, n = 6 in AH+L-NAME group; Fig A in S4 Fig), diastolic blood pressure (n = 12 in AH group, n = 6 in AH+L-NAME group; Fig B in S4 Fig). Foot blood flow monitored in vivo by LDI in each group of diabetic mice. Blood flow recovery was markedly improved in either aliskiren (25 mg/kg/day) treated mice (n = 12; Fig C and D in S4 Fig) or aliskiren and L-NAME (30 mg/kg/day) co-treated group (n = 6; Fig C and D in S4 Fig). AH represented aliskiren high dose (25 mg/kg/day); AH+L-NAME represented aliskiren high dose combined with L-NAME (30 mg/kg/day). *p < 0.05, **p < 0.01 compared with same group before treatment. (TIFF) [file pone.0136627.s004.tiff]

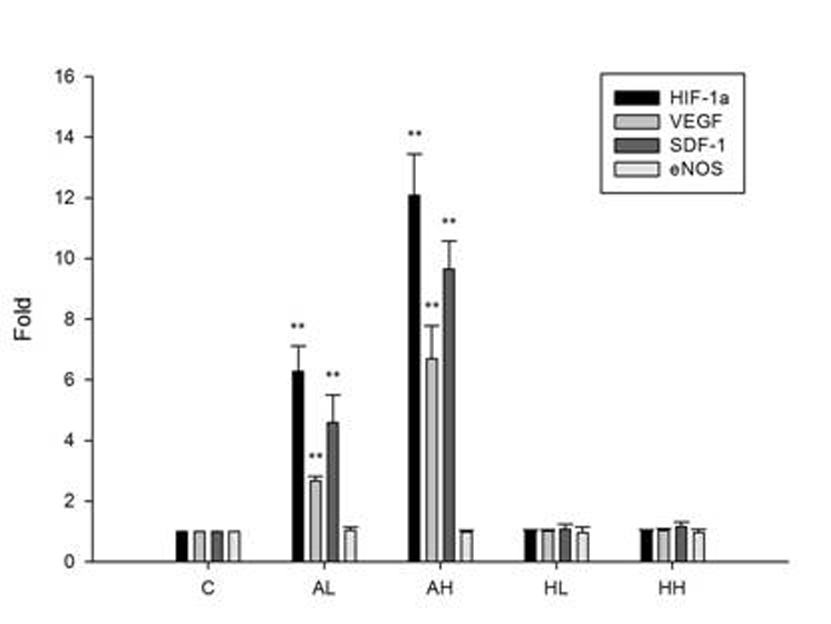

Supplement: S5 Fig — Quantitative RT-PCR for the effects of aliskiren on mRNA expressions in thigh muscles in diabetic mice with hindlimb ischemia (n = 6). Threshold cycle data were normalized to the internal standard, beta actin. C represents untreated diabetic mice (vehicle (PBS)-treated mice); AL represents aliskiren in low dose (5 mg/kg/day); AH represents aliskiren in high dose (25 mg/kg/day); HL represents hydralazine in low dose (2 mg/kg/day); HH represents hydralazine in high dose (10 mg/kg/day). *p < 0.05, **p < 0.01 compared with untreated diabetic mice (vehicle (PBS)-treated mice). (TIFF) [file pone.0136627.s005.tiff]

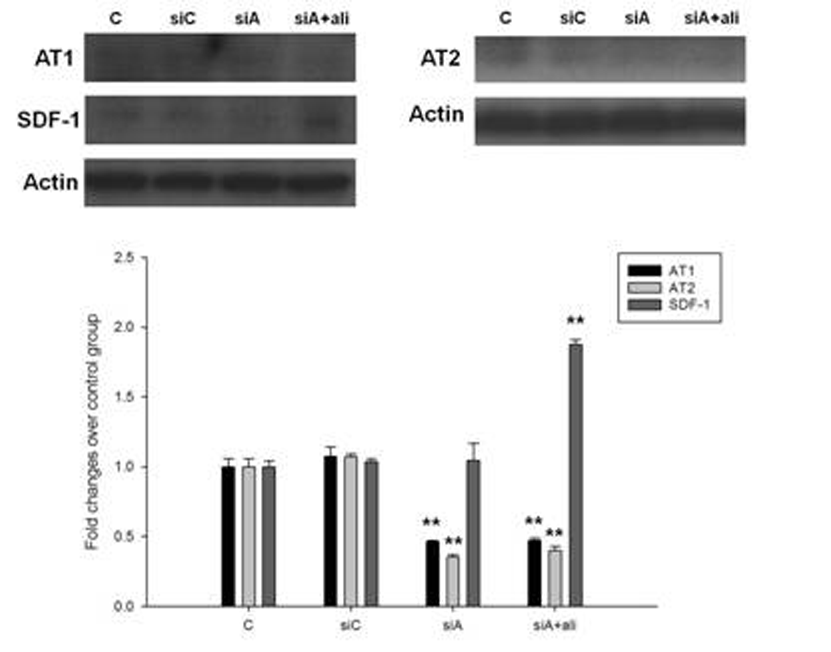

Supplement: S6 Fig — Western blot and statistical analysis of AT1, AT2, and SDF-1 expressions after slencing with AT1 and AT2 siRNA (n = 6). AT1 and AT2 siRNA did not abolished the effects of aliskiren (10 μM) on the expression of SDF-1 on EPCs from diabetic mice. C represents untreated cells; siC represents control siRNA; siA represents co-treated AT1 and AT2 siRNA; siA+ali represents combined treatment of AT1 siRNA, AT2 siRNA, and aliskiren (10 μM).*p < 0.05, **p < 0.01 compared with untreated cells. (TIFF) [file pone.0136627.s006.tiff]
